# Plant-Produced Vaccines for Protection from Human and Veterinary Coronaviruses

**DOI:** 10.3390/vaccines14080721

**Published:** 2026-08-21

**Authors:** Erin Egelkrout

**Affiliations:** Applied Biotechnology Institute, California Polytechnic University Technology Park, Bldg. 83, Suite 1D, San Luis Obispo, CA 93407, USA; erinegelkrout@appliedbiotech.org

**Keywords:** coronavirus, COVID-19, porcine epidemic diarrhea virus, plant-produced vaccine, oral administration, mucosal immunity, maize

## Abstract

Coronaviruses are responsible for numerous diseases causing substantial morbidity and mortality to both humans and animals. In particular, they have caused three significant outbreaks in humans including severe acute respiratory syndrome 1 (SARS-CoV-1), Middle East respiratory syndrome (MERS-CoV), and, most recently, the severe acute respiratory syndrome 2 (SARS-CoV-2) pandemic. Two relevant veterinary diseases are porcine transmissible gastroenteritis virus (TGEV) and porcine epidemic diarrhea virus (PEDV), which cause severe losses in the pork industry. While efficacious vaccines were developed in an unprecedented timeframe for COVID-19, vaccines have not been commercialized for other human coronaviruses and vaccines against animal coronaviruses have limited efficacy and logistical challenges in administration. There is a clear need for more efficacious vaccines with simpler methods of delivery and administration for both human and veterinary use. The production of subunit vaccines in plant systems holds great promise in addressing the current challenges and facilitate the preparation for future potential outbreaks and new viruses. This review will summarize the state of development of vaccines in plant systems including tobacco, rice, maize, and others.

## 1. Introduction

Coronaviruses are enveloped single-stranded positive-sense RNA viruses that target both humans and animals [1,2,3]. The *Coronaviridae* family can be divided into the four genera *Alphacoronavirus*, *Betacoronavirus*, *Deltacoronavirus*, and *Gammacoronavirus*. At 25–30 kb, the genome is relatively large. Three major structural proteins, the spike (S), the envelope (E), and the membrane (M), as well as the nucleocapsid (N) protein, are among the most important encoded genes relevant to vaccine design. Coronaviruses, along with rhinoviruses, are responsible for some cases of the common cold in humans, but three major outbreaks of more serious coronavirus disease have occurred in the last twenty-five years. These include severe acute respiratory syndrome (SARS-CoV) [4,5,6], Middle East respiratory syndrome (MERS-CoV) [7,8], and the more recent severe respiratory syndrome 2 (SARS-CoV-2) or COVID-19 disease [9,10,11,12] pandemic. For the two earlier outbreaks, numerous prototype vaccines have been developed but none have been commercialized. The COVID-19 pandemic precipitated a massive worldwide effort resulting in almost 200 prototype vaccines in an unprecedented timeframe, with 12 granted emergency use approval by the World Health Organization (https://covid19.trackvaccines.org/agency/who/) (accessed on 1 June 2026) and up to 50 vaccines approved in some countries [2,3]. Most of these vaccines have focused on the spike protein as an antigen, although virus-like particles (VLPs) including the other major structural proteins are also a promising approach. This research has also included the rapid expansion of new mRNA vaccine technology.

Numerous coronaviruses are also of high importance to veterinary practice and livestock health. Experience with earlier work on animal coronavirus vaccines contributed to the speed of development of COVID-19 vaccines [13]. Animal coronaviruses affect swine, poultry, cattle, cats, and dogs, among others. An effort has been directed to the development of vaccines for porcine transmissible gastroenteritis virus (TGEV) and porcine epidemic diarrhea virus (PEDV). Other common animal coronaviruses include avian infectious bronchitis virus (IBV), bovine coronavirus (BCoV), feline enteric coronavirus (FECV), feline infectious peritonitis virus (FIPV), canine coronavirus (CCoV), and canine respiratory coronavirus (CRCoV).

Despite the availability of vaccines for protection against coronavirus infection for both human and veterinary use, many vaccines are either still prohibitively expensive in some parts of the world, are not fully effective, or are inconvenient to implement. Thus, there is still widespread interest in the development of less expensive coronavirus vaccines that are simpler to distribute and administer. The production of vaccines in various plant systems has been an area of active study for decades with some recent successes. This review will summarize recent work specifically targeted at the development of plant-produced vaccines against coronaviruses for both human and veterinary use. In particular, it will also highlight work for both a human and an animal coronavirus demonstrating the potential of maize to serve as an ideal platform for the production of low-cost, heat-stable, orally delivered vaccines, and other pharmaceuticals.

## 2. Human Coronaviruses Overview

There have been three major outbreaks of coronaviruses infecting humans starting in 2002. The first (SARS-CoV-1) started in Guangdong Province, China and resulted in infection of 8098 people and 774 deaths (https://archive.cdc.gov/www_cdc_gov/sars/about/fs-sars.html (accessed on 1 June 2026)). The second, MERS, originated in camels and was first identified in Saudi Arabia in 2012 and has resulted in approximately 2628 cases and 948 deaths (https://www.emro.who.int/health-topics/mers-cov/mers-outbreaks.html (accessed on 1 June 2026)). For SARS-CoV-1, there have been some studies towards the development of a plant-produced vaccine and, for MERS, the expression of a DPP4-Fc fusion potentially preventing infection has been reported, but these have not progressed beyond the preclinical phase. In December of 2019, a new beta coronavirus, SARS-CoV-2, emerged in Wuhan, China and quickly spread, becoming a worldwide pandemic by March of 2020. The current WHO estimates are that 7,114,356 deaths can be attributed to COVID-19 (https://data.who.int/dashboards/covid19/deaths (accessed on 1 June 2026)). The COVID-19 pandemic resulted in an unprecedented effort to develop new vaccines in record time, including many prototype vaccines produced in plants from multiple groups around the world, several of which have progressed to clinical trials.

## 3. Severe Acute Respiratory Syndrome CoV-1 (SARS-CoV-1)

Three studies have attempted to produce prototype vaccines for protection against SARS in plants. Pogrebnyak et al. [14] expressed the N-terminal fragment (aa14–714) of the S1 domain of the spike protein in tomato and low-nicotine tobacco. Mouse immunization studies showed an increased fecal IgA, but not sera IgG, after the oral administration of tomato as 500 mg lyophilized fruit. Parenteral priming with tobacco derived protein also elicited IgG, but only after a booster dose of S peptide. No increase in IgA levels in sera was detected. In another study [15], a partial spike protein (aa1–658) was expressed in both the nucleus and chloroplast using tobacco and lettuce with confirmation by a Northern and Western blot but no animal efficacy studies. A study from Zheng et al. [16] focused on increasing the expression and showing that the p19 silencing suppressor enhances the transient expression of the SARS-CoV nucleocapsid N protein in *N. benthamiana* with an accumulation of up to 79 µg/g fresh wt. The intraperitoneal vaccination of mice with four doses of 2–4 µg elicited high IgG levels, with IgG1 10 fold higher than IgG2. The expression of IFN-ɣ and IL-10 also increased, but not IL-2 or IL-4.

## 4. Middle East Respiratory Syndrome (MERS CoV)

Although there is work on vaccines against MERS produced in non-plant systems, to our knowledge, work on plant-produced vaccines against MERS expressing an antigen from the virus has not been published. Given that numerous other coronavirus antigens have been successfully expressed in plants, this is likely largely due to a lack of a sense of urgency leading to challenges in obtaining funding. However, Planet Biotechnology has reported the expression in tobacco of a DPP4-Fc fusion that may prevent infection [17]. While this may not be strictly considered a vaccine, the anti-infective properties of this immunoadhesin protein are worth noting.

## 5. Severe Acute Respiratory Syndrome CoV-2 (SARS-CoV-2/COVID-19) Overview

Many attempts at the production of vaccines for protection against SARS-CoV-2 in plants have been made. This work from many groups worldwide in tobacco, rice, and maize is summarized in Section 5.1, Section 5.2, Section 5.3, Section 5.4, Section 5.5 and Section 5.6 below. Table 1 highlights the registered clinical trials from three major groups discussed below that have developed candidates that have progressed to this stage: Medicago in Canada, Baiya Phytopharm in Thailand, and Kentucky Bioprocessing in the United States.

### 5.1. The Medicago Vaccine

The Medicago vaccine is a virus-like particle (VLP) consisting of the prefusion-stabilized full-length spike protein produced by transient transfection in tobacco. The definition of VLP can be controversial and has been covered in several reviews, but, in some analyses, the fundamental characteristic has been described as the lack of a viral genome and infectivity, along with a distinctive viral appearance by electron microscopy [23,24,25]. Self-assembly into a highly organized structure is also key. At least one analysis defines VLPs as “structures made up of one or more different molecules” [25]. While the VLP formation for SARS-CoV-2 is usually considered to include additional viral proteins such as the M and E proteins, the spike protein alone was considered in this case to form this structure, although supporting data has not been published. It was successful in human clinical trials and was approved for use in Canada as Covifenz. This was a major milestone in the field as the first example of a plant-produced vaccine with regulatory approval for human use, but it was not approved for general use by the World Health Organization. Multiple commentaries have stated that this was largely due to the connections to the tobacco industry [26,27,28]. However, despite the substantial investment by the Canadian government, Medicago did clearly have challenges scaling up production and the efficacy of the Covifenz vaccine was not as high as the injected mRNA vaccines (https://thelogic.co/news/medicago-struggles-with-vaccine-production-as-it-hunts-for-a-new-minority-owner/ (accessed on 15 May 2026)). Although the Japanese company Mitsubishi Tanabe Pharma Corporation had acquired 60% ownership in Medicago in 2013, in 2022, Mitsubishi Tanabe bought out Philip Morris’ remaining ownership, which eventually led to the closure of Medicago and the discontinuation of the vaccine also due to the changing vaccine market and lack of commercial viability. However, a group of former employees are continuing the development of this technology in a new company named Aramis (https://aramisbiotechnologies.com (accessed on 15 May 2026)). The development of this vaccine resulted in multiple publications as summarized below.

It should be noted that, due to the unprecedented challenges and urgency of the SARS-CoV-2, the development of vaccines and the publication of work, in some cases, did not proceed in the usual expected manner, with human trial data published before animal trials or toxicology studies. The N. benthamiana-produced S protein VLP without an adjuvant or with AS03 or CpG1018 was tested in macaques, and the best results in the elicitation of humoral and cell-mediated response including the production of IL-2 and IL-4 in CD4 T cells were observed after the booster and with the AS03 adjuvant [29]. The vaccine protected the NHP from challenge as measured by the lower viral replication in bronchoalveolar lavage, the reduction in infected cells, and the reduced proinflammatory cytokines, with no evidence of vaccine-enhanced disease. Another study [30] found no evidence of negative effects on fertility, reproductive performance, or embryo–fetal development in Sprague–Dawley rats.

In June 2021, Ward et al. [18] reported interim safety and immunogenicity for a Phase I trial with adults 18–55 years of age after a prime and booster intramuscular injection at three different doses (3.75, 7.5, and 15 µg) 21 days apart either unadjuvanted or with the adjuvant AS03 or CpG1018. The vaccine was generally well-tolerated in all formulations without a dose effect. The inclusion of an adjuvant increased neutralizing antibody titers and the titer increased after the second dose to a level significantly higher than that observed in convalescent sera. The induction of interferon-ɣ and IL-4 was also observed. A further analysis of the Phase I trial in healthy adults [19] including the durability and cross-reactivity of the immune response was subsequently reported. Six months after the second dose, neutralizing antibodies were still observed in 95% of individuals, and INF-ɣ and IL-4 were still observed in over 90% of individuals. Pseudovirus and live-virus neutralization assays indicated a broad cross-reactivity against multiple variants including Alpha, Gamma, Delta, and, to a lesser extent, Omicron, at day 42 and day 201.

An interim analysis of the Phase 2 portion of the Phase 2/3 trial NCT04636697 was reported by Charland et al. [22]. This part of the study included 573 individuals distributed between healthy adults, older adults, and adults with comorbidities randomized 5:1 for two intramuscular doses of 3.75 µg CoVLP-AS03 or placebo three weeks apart to assess the safety and immunogenicity, neutralizing antibody titers, cross-reactivity to variants, and IFN-ɣ and IL-4 response. The vaccine was well-tolerated with mild to moderate transient adverse events, which were somewhat less frequent in older adults and adults with comorbidities. Seroconversion in 98% of individuals was observed three weeks after the second dose, with neutralizing antibody levels 10× that in convalescent sera. There was a varied but substantial cross-reactivity to the Alpha up to Omicron variants at six months after the second dose. An IFN-ɣ and IL-4 response was still detectable in 88% of participants 211 days after the second dose, which constitutes a Th1-biased response. Hager et al. [21] reported the results of a Phase 3 multicenter clinical trial (NCT04636697) with ca. 24,242 adults 18 years or older receiving a prime and booster dose 21 days apart with either CoVLP + AS03 or placebo. The efficacy ranged from 69.5% against any symptomatic COVID-19 to 78.8% against moderate-to-severe disease, with tolerable mild or moderate adverse events.

Kaplonek et al. [20] re-examined the Phase I trial data comparing the humoral immune response elicited by the Medicago vaccine without an adjuvant or with the AS03 adjuvant up to six months after the second dose. They found a strong IgG1 antibody response, FcɣR binding, and antibody effector function. The neutralizing antibody levels were declining but detectable FcɣR2A and effector functions including opsonophagocytosis were still observed. Another study aimed to examine the effect of AS03 on the memory B cell response to distinguish the development of antigen-specific B-cell clones with different specificities or clones inherently producing broadly cross-reactive antibodies [31]. This study also compared the Medicago plant vaccine with mRNA-1273 and found a better correlation between CD4^+^ T cells at d42 and memory B cells at six months with the Medicago vaccine than with mRNA-1273. Changes in the B-cell response and somatic hypermutations over six months suggest an increase in the neutralization breadth and progressive maturation of the B-cell response.

### 5.2. Baiya Phytopharm

Baiya Phytopharm based in Thailand has explored two vaccine candidates based on the receptor binding domain (RBD) portion of the SARS-CoV-2 spike protein. In an early report from Siriwattananon et al. [32], the RBD protein fused with the human IgG1 Fc fragment was expressed in *N. benthamiana*, protein A purified, and shown to retain the binding to the human ACE2 receptor produced in HEK293 and CHO cells. Immunization by prime and boost intramuscular injection with an alum adjuvant of mice and cynomolgus macaques resulted in high neutralization titers, mixed Th1/Th2 cytokines, and a T-lymphocyte response. In a follow-up, a direct comparison of multiple commercially available adjuvants including alum, MF59, mPLA, and poly(I:C) was performed [33] All adjuvants elicited the induction of IgG and neutralizing antibodies, but only two, mPLA-SM and poly (I:C), allowed a balanced IgG1 and IgG2a (Th2/Th1) response. Poly(I:C) also enhanced specific IFN-ɣ cellular immunity. Rattanapisit et al. prepared RBD-Fc fusions for the Alpha and Beta SARS-CoV-2 variants to confirm the binding to the ACE2 receptor and to anti-spike monoclonal antibodies [34]. Binding to monoclonal antibodies CR3022, B38, and H4 was tested, with some reduced binding affinity for the Beta variant RBD construct relative to the Alpha variant construct. A subsequent more extensive study in macaques with the Baiya SARS-CoV-2 Vax 1 prototype [35] tested the immunogenicity after injection at days 0, 21, and 133. A neutralizing antibody response against SARS-CoV-2 variants Alpha, Beta, Gamma, Delta, and Omicron was observed. A subsequent study [36] continued the detailed analysis for the cross-protection in macaques against variants Alpha, Beta, Gamma, Kappa, Delta, and Epsilon. This prototype was also found to elicit a neutralizing antibody response and protection from challenge in hamsters after immunization with two intramuscular doses (10 µg) of Baiya SARS-CoV-2 Vax 1 [37]. Shanmugaraj et al. [38] further confirmed the immunogenicity and safety of the Baiya SARS-CoV-2 Vax 1 in macaques, but also added efficacy in the K18-hACE2 mouse model as well as toxicity in Wistar rats. The mouse model with hACE2 makes a challenge possible, and a reduced viral load after a challenge three weeks post immunization was observed. There were also no safety concerns observed in the rats.

While the previous work included only the RBD domain of the spike protein, Panapitakkul [39] pursued the production of the longer SARS-CoV-2 S1 domain as an Fc fusion. The immunization of mice resulted in an increase in IgG1 relative to IgG2. Finally, a SARS-CoV-2 recombinant RBD-Fc second-generation vaccine designated Baiya SARS-CoV-2 Vax 2 designed to target newer variants was tested with a focus on the comparison of a newer TLR7/8 agonist adjuvant, 3M-052, to alum [40]. Protection was demonstrated after a two-dose immunization and viral challenge in humanized K18-hACE2 mice. This candidate supported a higher anti-RBD neutralization titer against multiple variants in both mice and monkeys relative to Baiya SARS-CoV-Vax 1. The immunization of cynomolgus macaques was safe and well-tolerated and toxicity studies in rats showed safety. The Baiya vaccine candidate progressed to human clinical trials, but the results have not been fully published other than some preliminary safety data posted on the company’s website or added to the clinical trials registry.

### 5.3. Kentucky Bioprocessing

Kentucky Bioprocessing has developed a purified SARS-CoV-2 vaccine candidate produced in tobacco consisting of the spike protein receptor binding domain (RBD) fused to a human IgG1 Fc domain [41]. Conjugation to a variant of the tobacco mosaic virus creates a nanoparticle formulation similar to a virus-like particle (VLP) produced in tobacco. The candidate binds the ACE2 receptor and SARS-CoV-2 neutralizing antibodies. A strong antibody response was elicited in mice, although the balance of the Th1 and Th2 response depended on the inclusion of the CpG adjuvant. This prototype vaccine was used in a human clinical trial with some demographic distribution and adverse event results posted to the clinical trial registry, but the company has been undergoing reorganization, and further progress has not been reported.

### 5.4. iBio

Another series of tobacco-produced SARS-CoV-2 prototype vaccines has been developed by iBio in Bryan, Texas, designated as iBio-200, iBio-201, and iBio-202. These target either the spike protein or, for iBio-202, the nucleocapsid protein, and incorporate a proprietary lichenase carrier molecule called LicKMTM. These have progressed through substantial preclinical testing (https://ir.ibioinc.com/news-events/press-releases/detail/135/ibio-to-advance-covid-19-lickm-subunit-vaccine-candidate-ibio-201 (9 September 2020)) and IND submission but iBio has refocused to antibody production and does not appear to be currently pursuing these candidates.

### 5.5. Other Plant-Produced Prototype Vaccines

Several publications from a group based at Akdeniz University and colleagues in Turkey report prototype vaccines based on the expression of the spike protein RBD. Mamedov et al. [42] describe the expression of the nucleocapsid protein N up to 45 mg/kg in *N. benthamiana*, as well as the co-expression of RBD to prepare an antigen cocktail. The purification of the RBD and N antigens, the analysis of size by gel filtration, and stability studies preceded animal studies. Mouse immunization with N or N + RBD at 5 µg elicited high antibody titers only with the cocktail. A micro neutralization test demonstrated neutralizing activity with RBD + N at a titer of 1:128. In a companion publication [43], the same group produced glycosylated RBD (gRBD) and a variant deglycosylated by the co-expression with Endo H (dRBD). The expression was targeted to the ER and levels of at least 42 mg/kg fresh wt. were observed. This was followed by anti-FLAG column purification and the demonstration of specific binding to the ACE2 receptor in vitro. Stability testing showed better stability for the deglycosylated form. The immunization of mice with 5 ug gRBD or dRBD elicited high antibody titers and neutralizing activity for both the deglycosylated and glycosylated form, although the neutralization titer was higher on immunization with the deglycosyated RBD. In a subsequent work [44], this group demonstrated that plant-produced glycosylated and nonglycosylated RBD and cocktails with the N protein can neutralize the emerging variants Delta and Omicron. Mice were immunized twice with 5 µg with glycosylated RBD, deglycosylated RBD, or glycosylated RBD + N. Higher titers were observed with the cocktail and, while the titer for the neutralization of the Delta variant was comparable to that for the original Wuhan strain, the neutralization of the Omicron variant was about four-fold lower. The authors also argue that their specific RBD fragment with an even number of cysteines allows better folding and results compared to other versions with an uneven number of cysteines.

Maharjan et al. [45] used glycoengineered *N. benthamiana* to express the RBD of SARS-CoV-2 spike by transient expression in a TMV-based vector with levels up to 92 mg/kg fresh wt. This candidate supported the elicitation of humoral immunity and neutralizing antibodies in mice and a comparison of different adjuvants showed the best results with AddaVax, although a high IgG1/IgG2 ratio suggests a biased Th2-type response. Focus reduction neutralization (FRNT50) tests with the live virus in VeroE6 cells were also performed. Mardanova et al. [46] achieved the transient expression of a flagellin-RBD fusion using the pEff vector at 110–140 ug/g fresh wt. without codon optimization towards developing an intranasal vaccine but this did not include detailed efficacy or animal studies. An extensive study to determine the relative role of N-glycosylation in the expression, folding, and efficacy of RBD expression in plants was carried out by Shin et al. [47]. They performed transient expression of the variants in RBD length and post-translational modifications and found a truncated version consisting of Arg319-Leu533 (RBD-215) to be the most effectively expressed. As in some of the previous work, the sequence was chosen to include an even number of cysteines. They found complex glycosylation at two sites on the expression in ΔXT/FT *N. benthamiana*. They suggest that the glycan composition is important for folding, but not as much as for expression and function. However, the mutation of glycosylation sites resulted in the loss of detectable expression. They tested ER retention in combination with co-expression with the folding chaperone calreticulin (CRT) or other CRT family proteins. The inclusion of CRT mediated ER-retention in protein bodies but may not substantially improve folding. The co-infiltration of N-glycan processing inhibitors did not alter the expression or secretion of RBD-RFP.

A number of groups in South Africa, both public and private (Cape BioPharms), have published extensive work on potential plant-produced SARS-CoV-2 vaccines. Although the goal was more to develop a serological assay using plant-derived recombinant viral proteins, Makatsa et al. [48] expressed the S1 and RBD portions of the spike protein in *N. benthamiana* with fusion to a rabbit IgG Fc tag. The plant-produced antigens detected SARS-CoV-2-specific antibodies in patient serum with a good correlation with existing assays for IgG, IgA, and IgM. There has been an extensive effort to bioengineer expression in tobacco for better maturation, folding, cleavage, stability, and functionality of recombinant proteins with the transient co-expression of human chaperone proteins and furin. This group has developed a specialized *N. benthamiana* expression platform called NXS/T Generation^TM^ to optimize recombinant protein folding and glycosylation. Margolin et al. [49] aimed to express the full spike protein antigen lacking the transmembrane and cytoplasmic domains in a form closer to the native conformation than existing RBD prototypes by including a GCN4 fibritin trimerization domain and an optimized furin cleavage site. At this time, further stabilizing mutations of the spike protein were not included. The co-expression of the chaperone calreticulin substantially improved expression. The co-expression of human protease was also required for efficient cleavage, but this did not appear to impair endogenous degradation and showed no clear improvement in expression. The immunogenicity in mice after three doses with 3 µg and allohydrogel adjuvant was tested with the elicitation of binding antibodies plateauing after the first boost and the induction of IFN-ɣ secretion and high titers of neutralizing abs. In a subsequent publication [50], the co-expression of the chaperones calnexin or calreticulin combined with glycoengineering including the co-expression of LmSTT3D, RNAi to ablate beta-N-acetylhexosaminidase, and expression in ΔXF *N. benthamiana* to prevent plant specific glycans was tested with a spike construct HexaPro with six proline mutations for stabilization in the prefusion conformation. An analysis of site-specific glycans showed mostly oligomannose-type N-glycans. The immunization of hamsters (5 µg) in comparison with the mammalian-produced HexaPro antigen showed the elicitation of neutralizing antibodies against matched and heterologous strains, but the levels were lower for the plant-produced antigen. Challenge with the Delta variant showed vaccine protection against weight loss, a reduction in viral load, and reduced lung histopathology. O’Kennedy et al. [51] developed a Beta variant S protein VLP vaccine candidate in ΔXT/FT *N. benthamiana* and tested this with three different adjuvants in New Zealand white rabbits, also in comparison to the S protein from the early Wuhan strain. The existing SEPIVAC SWE adjuvant was superior to the other two tested. The Beta variant vaccine candidate elicited robust neutralizing antibody responses after booster vaccination, including against heterologous Delta and Omicron variants, but less cross-neutralization was observed after vaccination with the Wuhan strain candidate. A recombinant Beta-variant full-length spike protein VLP was also tested in golden Syrian hamsters, resulting in the neutralization of both the homologous Beta variant and heterologous Delta and Omicron variants, as well as protection against challenge as measured by reduced viral RNA, weight loss, and lung damage [52].

Another approach to enhancing the durability of protection by including additional viral antigens was taken by Jung et al. [53]. The transient expression in *N. benthamiana* was used to produce and test the full-length S protein with or without the envelope (E) and membrane (M) proteins, although the expression was higher with the S protein alone (23 vs. 8 mg/kg fresh wt.). An extensive analysis was performed to confirm the higher-order structure and binding to patient sera, but the goal was more reagent production for study than vaccine development. Moon et al. [54] also demonstrated the transient expression of M, N, and E but not spike proteins relevant to VLP production.

In an effort to optimize the formation of the prefusion-stabilized S protein trimer, Song et al. [55] used the transient expression of the full-length S protein lacking the transmembrane domain and C-term cytoplasmic tail but including 3P proline substitutions for the stabilization and mutation of the furin cleavage site. They compared the fusion to different trimerization motifs including the foldon of bacteriophage T4 fibritin, mouse Coronin 1A (mCor1), and BiP. Expression up to 106 µg/g fresh wt. was observed with mCor1 and the co-expression of calreticulin (CRT). Mouse immunization (3X with 1–30 µg and alum adjuvant) elicited the production of both S1 and S2 subunit antibodies and neutralizing antibodies. This was only with the highest dose after the first boost, but with all doses after the second boost. The immunization of hACE2 transgenic mice elicited neutralizing antibodies and protection from challenge as well as some neutralization against newer variants.

While the efforts at the development of a plant-based vaccine have generally been safe and well-tolerated in animals, Park et al. [56] performed an extensive toxicity study involving repeated intramuscular injection to evaluate the safety of a purified tobacco-produced vaccine in rats. The vaccine consisted of the spike protein RBD domain adjuvanted with the TLR4 agonist. Antibodies were detected up to one month after vaccination. A panel of numerous measures including body weight, food consumption, organ weight, hematology, serum biochemistry, and histopathology were assessed and did not suggest major concerns.

The previous work focused on parenteral administration but there are potential advantages to mucosal delivery as mucosal tissues are the site of the pathogen entry. This may be particularly relevant to its use as a booster in situations where parenteral prime immunization has already been achieved in a substantial portion of the population. Kim et al. [57] used a novel *N. benthamiana* plant expression system Platform CTB-Fc (PCF) to test a mucosal vaccine platform incorporating the fusion of the spike protein antigen to molecular adjuvants IgG-Fc and Cholera toxin B (CTB). IgG-Fc targets the vaccine to IgG receptors on antigen-presenting cells and CTB targets cellular gangliosides in the mucosae. The vaccine is highly immunogenic on its use as an intranasal prime dose in mice and elicits neutralizing systemic and mucosal antibodies and memory T cells in the lungs, as well as a relatively balanced Th1 and Th2 response. It was shown that the vaccine can be aerosolized without a loss of activity. There was some indication of protection from challenge but future work with a larger number of mice and the hACE2 mouse strain is planned.

The previously described work focused on production in tobacco, but ideally plant-produced vaccines would be made in an edible species for potential oral administration. Several publications describe the production of SARS-CoV-2 antigens in rice. Saba-Mayoral et al. [58] demonstrated the stable transformation of rice callus with the S protein RBD under the control of a constitutive ubiquitin promoter or endosperm-specific barley D-hordein promoter and binding to the ACE2 receptor by ELISA. The best expression level was 6.88 µg/g fresh wt. with the ubiquitin promoter and, in the T1 seed, the expression was 5.31 µg/g dry wt. In a subsequent publication [59], the same group focused on the glycan structure for all uses of the plant RBD expression in rice callus. In this study, levels up to 3.5 µg/g fresh wt. were achieved. One of two potential N-glycan sites was not utilized and the other showed a mix of complex-type N-glycans. This differs from the mix of glycans in *N. benthamiana*. In a more extensive study of a rice-derived vaccine candidate, Song et al. [60] developed vector pCAMBIA 1300Gt1-S1 with the S1 subunit under the control of a rice-seed-specific glutelin promoter or constitutive actin promoter and observed the expression in seed of up to 282 µg/g dry wt. or 6.3% TSP with the glutelin promoter. Surprisingly, the actin promoter did not support detectable expression. A high affinity for the ACE2 receptor in vitro was demonstrated, with some variation in the affinity for the deglycosylated and glycosylated form depending on the ACE2 concentration. The immunogenicity by intramuscular injection (3X) in mice with various adjuvants led to IgG, IgG1, and a neutralizing antibody response. A better mixed Th1/Th2/Th17 cytokine response with the FliC adjuvant and an increase in IFN-gamma, IL-4, and IL-17A CD4^+^ T lymphocytes was found. This study lays the groundwork for future planned studies on oral immunization.

Lettuce is another candidate species for the potential expression of edible plant-produced vaccines. Singh et al. [61] showed the expression at 11.8 mg/g dry wt. of a full-length spike protein fusion with the cholera toxin B subunit (CTB) under the control of a psbA promoter in a specialized chloroplast system with marker excision. It should be noted that no glycosylation is expected in chloroplasts. The interaction with the ACE2 receptor was demonstrated by the inhibition of enzyme activity. This candidate is envisioned as a potential heat-stable booster vaccine, potentially as a dried powder, but further studies have not been published.

### 5.6. Production in Maize

Most of the existing prototype SARS-CoV-2 vaccines are produced in tobacco. This requires purification away from other toxic compounds and is not amenable to oral delivery. In addition, it usually requires cold chain and traditional parenteral administration relying on trained medical personnel and mobilization and the disposal of additional materials such as needles and syringes. An alternative production system in maize has been studied that can overcome these restrictions and is amenable to the rapid scale-up and production of very large numbers of doses without impinging on land use for food or feed [62,63,64]. Recombinant proteins in maize grain are highly stable and, with targeting of expression to the seed, can be stored in grain for years. This allows the just-in-time processing into ground flour, that can be orally delivered in resource-challenged areas. In addition, the use of enhanced promoters targeting expression to the seed embryo allows a combination of a high initial expression with further improvements by separating the germ from the endosperm, as well as traditional breeding techniques to achieve levels of several grams of recombinant protein per kg of seed material. Several studies at Applied Biotechnology Institute have demonstrated a proof of concept for efficacious maize-produced vaccine candidates for hepatitis B [65,66,67,68,69] and Valley fever (Coccidioidomycosis) [70] in mice, as well as for veterinary vaccine candidates, as discussed below.

To show that this approach can be used for SARS-CoV-2, several constructs were prepared to express the spike protein in the maize embryo. High levels of expression of the spike protein from the earliest Wuhan Hu-1 strain in maize were achieved [71] and maize grain has been used in a pilot mouse-feeding study (unpublished). While much more work is needed to optimize the response, this preliminary data provides support that an orally delivered maize-produced candidate may be a viable method for the immunization against SARS-CoV-2 or future emerging viruses. However, for better protection, the most relevant variant can be used to make VLPs, which should provide protection against novel variants. The administration of a VLP is likely to provide even stronger protection and a greater cross-reactivity to other variants. In addition, we subsequently found that the fusion of the spike protein to the carrier protein E. coli heat labile enterotoxin B (LTB) resulted in an increased formation of high-molecular-weight multimers consistent with the formation of trimers, and this should increase the efficacy in future experiments [71].

## 6. Veterinary Coronavirus Vaccines Overview

While there are numerous coronaviruses that infect various animal species, most of the work on potential plant-based vaccines has focused on two viruses that infect swine, porcine transmissible gastroenteritis virus (TGEV), and porcine epidemic diarrhea virus (PEDV). Both diseases cause severe diarrhea and high mortality in young piglets. TGEV was first identified in the United States in 1946, although it is now less of a concern. PEDV was first identified in Europe in the early 1970s and has since spread to China and the United States to become one of the most challenging pathogens in swine production. In both cases, there are existing vaccines that are not fully efficacious and there is a pressing need for more effective vaccines with a simpler method of administration such as the addition to standard feed. Prototype vaccines for TGEV and PEDV have been expressed in a variety of species including tobacco, potatoes, and maize. As these diseases are most severe in young piglets, the goal is often vaccination for the development of lactogenic immunity that can be passed to piglets through milk and colostrum. In addition to TGEV and PEDV, there are a few reports of prototype vaccines for other veterinary coronaviruses including avian infectious bronchitis virus (IBV) and porcine delta coronavirus (PDCoV).

## 7. Porcine Transmissible Gastroenteritis Virus (TGEV)

### 7.1. Vaccine Candidates in Tobacco, Arabidopsis, and Potato

Several studies have attempted to produce plant-produced vaccines in Arabidopsis, tobacco, and potato. Gomez et al. [72] had difficulty expressing the full-length spike protein but expressed the S1 subunit N-terminal domain of TGEV in potatoes. The expression was up to 0.07% TSP by ELISA but not detectable by Western blot. Intraperitoneal administration to mice led to the elicitation of S-protein IgG. The oral feeding of potato tubers also elicited the development of serum antibodies, but no induction of neutralizing antibodies was observed. Antigen antibody complexes were demonstrated by immunoprecipitation with ^35^S-methionine-labelled protein. In another report, the same group also expressed the N-terminal aa 1–750 in Arabidopsis and tobacco and did indicate some neutralizing antibody activity was detected [73].

To improve the expression, Tuboly et al. [74] prepared three different constructs: one with a stronger promoter, one with codon optimization, and one fused to alfalfa beta amylase, leading to the expression in *N. tabacum* estimated at 0.1–0.2% TSP. Pigs were immunized by injection with the extract and all groups showed a strong TGEV-specific immune response and some elicitation of neutralizing antibodies. It appears oral immunization was a long-term goal but has not been fully pursued. In another 2013 report [75], Ahn et al. prepared three constructs with a 700 bp fragment of TGEV S under the control of three different promoters for expression in potatoes. No abnormalities in the potato morphology or tuber components were observed and levels of 55–163 ng/g fresh wt. or 0.015% TSP by ELISA were obtained with the patatin promoter. In a mouse study, 25 µg potato-produced antigen was used as a prime immunization by oral gavage, followed by a boost with an intraperitoneal injection of 5 µg E. coli-produced TGEV S. A strong serum IgG response and small but significant fecal IgA response was detected.

### 7.2. TGEV Prototype Vaccines in Maize

As indicated above, maize grain has the potential for the high level production of viral antigens that can be orally delivered. Proof of concept has been shown that a potential TGEV vaccine produced in maize is efficacious and provides protection from challenge on oral delivery to pigs. In two initial studies, the maize-codon-optimized TGEV S protein with a barley alpha amylase cell wall signaling sequence was produced [76,77]. The oral feeding of 10-day old piglets (2 mg) followed by a challenge with the Purdue TGEV strain showed protection from the development of symptoms and a reduced clinical severity index relative to control corn feeding. In a further study of the efficacy and function of maize-produced S protein, Lamphear et al. [78] used fractionation to determine the recombinant protein levels in grits, germ, and bran. Studies over 10 months with uncontrolled storage or storage at 4 °C or 10 °C showed good stability. Oral feeding TGEV corn to piglets followed by exposure to the TGE whole virus at day 29 showed the elicitation of neutralizing antibodies. Different doses and consecutive administration for 4, 8, or 16 consecutive days were also tested, with 4 consecutive days showing the best results. In a follow-up study on lactogenic immunity [79], S protein expression at 13 mg/kg grain was achieved, and an antigen dose of 20–30 mg was used. Two oral doses of the commercial modified live TGEV vaccine were administered on the day of breeding and at 102 days before farrowing, followed by an intramuscular injection with commercial modified live TGEV vaccine at 88 days before farrowing. Subsequently, at 35 days and 14 days before farrowing, the gilts were administered the oral corn-produced vaccine or controls. The oral vaccination of gilts with the corn-produced vaccine boosted the neutralizing antibody levels in animals’ serum, colostrum, and milk.

## 8. Porcine Epidemic Diarrhea Virus (PEDV)

### 8.1. PEDV Candidate Vaccines in Tobacco

Many potential plant-produced vaccines for protection against PEDV have been produced in tobacco, although we have reported a promising candidate produced in maize, and a few other species including potato, rice, lettuce, and duckweed have been studied. Most of these candidates focus on the expression of the spike protein and, in particular, the amino terminal S1 domain. A few reports have examined the potential of other domains such as S2.

In one of the earliest reports, Bae et al. [80] expressed the neutralizing epitope of the PEDV spike protein in transgenic tobacco, and the ELISA showed levels of 8–20 µg/g wet wt. Mice were immunized by parental injection with extracted protein, and the immunogenicity of the antigen was tested by the plaque reduction neutralization titer (PRNT). Oral feeding also induced a systemic and mucosal IgA immune response against the antigen and, again, the induced antibodies inhibited the virus by PRNT. Antigen-specific lymphocyte proliferation also increased in response to the feeding of the tobacco-produced vaccine candidate.

In one of the first of a series of studies from a group based at Chonbuk National University in Korea and collaborators, Kang et al. [81] used a TMV vector to express the codon-optimized CO-26K equivalent (COE) in tobacco, which contains critical epitopes for protection. Expression up to 5.0% TSP and 30-fold improvement with codon optimization was achieved. No animal trials were included but a goal of the immunization of sows for lactogenic immunity was presented. In a follow-up from the same group [82], it was shown that the COE can be expressed in a tobacco strain without nicotine, potentially reducing the concerns regarding toxins for production in tobacco. In a further enhancement, this group expressed the S protein COE with an N-terminal fusion to LTB [83]. A GM1 ganglioside binding assay showed strong affinity of the LTB-COE fusion for this intestinal membrane receptor, but no animal studies or direct comparison to the construct without LTB were included. Huy et al. [84] later expressed the S1D fragment (aa636–789) with and without fusion to the CTB carrier protein. Expression reached 0.04% TSP for S1D and 0.07% for CTB-S1D, which increased four-fold and seven-fold to 0.15% and 0.49% TSP, respectively, with ap19 co-expression to prevent silencing. The oral administration of the antigen to mice induced a serum IgG and IgA response. The antibody response was enhanced by antibody co-administration with additional bacterial cholera toxin (bCT) or a mutant version of the cholera toxin (rCTX).

In the first of a series of four studies from a group based in Vietnam, but with some overlap with the Korean group, Ho et al. [85] expressed a COE fusion with a C-terminal isoleucine zipper trimerization (GCN4pII) motif in *N. benthamiana*. Purification by immobilized metal affinity chromatography (IMAC) and confirmation of trimeric structure by SDS PAGE and BS3 cross-linking were performed. The subcutaneous immunization of mice with a prime and two booster doses of crude extract elicited a humoral immune response including IgG, IgA, and IgM, as well as a neutralizing antibody response. Tien et al. [86] expressed the COE as a fusion to the polymeric immunoglobulin G scaffold (PIGS) in glycoengineered ΔXT/FT tobacco with the co-expression of the J chain to enhance the proportion of dimers and polymers. Binding to C1q and surface antigen-presenting cells in vitro was demonstrated. Both the systemic and oral vaccination of mice with the COE and bacterial CT adjuvant elicited a humoral and cellular (B cell) immune response and oral administration elicited both IgG and IgA. To extend the prior work to testing in pigs, Ho et al. [87] prepared a COE-GCN4pII trimerization motif construct from a current Vietnamese genotype G2a strain and achieved expression in *N. benthamiana* at 2.01% TSP. Pregnant sows were immunized by parenteral administration. Both sows and five-day old piglets were tested for IgG, IgA, neutralizing Abs, and IFN-ɣ, and all increased with the administration of the plant-based vaccine to sows. Challenge experiments showed all piglets from the vaccinated sows survived. While most studies have used smaller portions of the N terminal half of the spike protein up to the S1 subunit, another study from this group [88] expressed the S2 subunit (aa730–1324) of the PEDV G2a strain with a fusion to the GCN4pII trimerization motif. Expression was reported to be 1.47% TSP, which was actually over 200-fold higher than the corresponding S1 GCN4pII construct. The purification was by IMAC and the characterization of oligomeric state was performed by size exclusion chromatography (SEC), but no animal studies were performed.

Sohn et al. [89] stably expressed the recombinant S1 protein as a fusion to porcine Fc (pFc2) to test a longer portion of the spike protein than in previous work. Glycosylation analysis was performed with endoglycosidase H. The vaccination of pregnant sows was performed to assess the development of humoral immunity to S1 in piglets, and the elicitation of a neutralizing antibody response in both sows and piglets was detected. A challenge study showed the protection of piglets from vaccinated sows.

### 8.2. PEDV Vaccine Candidate in Maize

A potential vaccine has been produced by Applied Biotechnology Institute in maize with the expression of the S1 subunit under the control of an embryo-specific maize globulin promoter and targeting to the endoplasmic reticulum. This candidate was shown to be efficacious in inducing neutralizing antibodies on the oral delivery of ground maize flour to pigs [90], although it should be noted that a response to the maize-produced vaccine relative to the control was not detected until after the challenge. This work was extended to demonstrate the passive transfer of immunity from vaccinated sows to piglets and the protection on challenge [91]. In this study, it was also suggested that protection may correlate with lower levels of granulocyte-macrophage colony stimulating factor. An additional construct with a fusion to the LTB carrier protein also increased the levels of higher-molecular-weight complexes consistent with the formation of trimers. The spike-protein–LTB fusion also showed an enhanced efficacy in eliciting the mucosal IgA relative to the spike protein alone when used for the vaccination of swine [71].

### 8.3. PEDV Candidate Vaccines in Other Species

In an earlier study, Kim et al. [92] showed the expression of the COE in potatoes under the control of the constitutive 35S promoter. PCR, northern and western blot analysis, and ELISA identified two relatively high expressing lines, but no animal studies were performed. Rice endosperm was used as an expression platform for a COE-LTB N-terminal fusion with the HMW Bx17 rice glutenin endosperm-specific promoter and rice actin first intron [93]. RT-PCR and protein analysis by western blot, ELISA, and GM ganglioside binding assay were performed to show expression at 1.3% TSP, but no efficacy testing in animals was pursued.

Two studies have attempted the production of PEDV spike protein in lettuce. In the first, Huy et al. [94] expressed a COE-LTB N-terminal fusion in lettuce. An analysis by Northern blot, Western blot, and ELISA was performed and the GM1 ganglioside assay showed a strong affinity of the LTB-COE fusion for the receptor, but no animal studies or direct comparison to the construct without LTB are described. In a subsequent study [95], a synthetic sCTB-sCOE construct under the control of the ubiquitin promoter was transformed into lettuce. Transgene integration and expression was confirmed with genomic PCR, Northern blot, and Western blot consistent with pentamer formation, but ELISA showed expression at 0.0065% TSP. Binding to GM1-ganglioside was demonstrated but no animal studies were performed. Ko et al. [96] attempted expression in transgenic duckweed and the optimization of different antibiotic levels for selection. The confirmation of transgene integration by PCR and RT-PCR and expression by immunoblotting was performed but no further efficacy studies or animal experiments. Huy et al. [97] demonstrated expression at 0.083% TSP in rice calli of sCOE with a fusion to the M cell targeting ligand (Co1) under the control of the Ramy3D promoter, which is inducible by sucrose starvation. After a glycosylation analysis using PNGase F, Endo F1, and Endo H, the rice callus extracts were orally administered to mice in 10 doses, eliciting an increase in serum IgG and fecal IgA, as well as an increase in IgG and IgA secreting cells by ELISPOT.

## 9. Avian Infectious Bronchitis Virus

While most of the work on veterinary coronavirus has focused on swine, a number of studies have explored potential vaccines for avian infectious bronchitis virus (IBV). This is a very contagious respiratory disease in chickens caused by a Gammacoronavirus with many regional variants. This high variability highlights the need for alternatives to the existing inactivated whole virus vaccine produced in eggs. In an early study, Zhou et al. [98] prepared transgenic potatoes with IBV S1 with expression up to 0.22% TSP. The immunization of both mice (three 5.72 µg antigen doses by gastric gavage of tuber extract) and chickens (three oral or intramuscular 28.6 µg or 57.2 µg doses) elicited neutralizing antibodies. The neutralization titer from chickens vaccinated orally was similar to that obtained with the commercial vaccine after the third dose. This study also showed protection from challenge. The same group then expressed the full-length spike protein in potato at up to 2.53 µg/g fresh wt. [99]. The oral and intramuscular immunization of chickens with three doses elicited neutralizing antibodies and protection from challenge. The secretion of IL-2 and T lymphocyte proliferation was assessed, and both were found to increase. In this study, the response by all measures was slightly better with intramuscular than oral administration.

In two more recent publications, Sepotokele et al. [100] used co-infiltration in *N. benthamiana* of the spike protein with the transmembrane domain and cytoplasmic tail replaced with the sequence from the Newcastle disease virus (NDV) glycoprotein and the NDV Matrix protein for a yield of 16.8 mg/kg of leaf material. They observed lower expression levels with the native modified IBV spike protein co-expressed with IBV M, E, and N proteins. The vaccine prototype was tested in chickens by intramuscular administration, and seroconversion in response to immunization was detected as measured by hemagglutination inhibition titers. In a follow-up publication [101], the authors use the previously described *N. benthamiana*-produced VLP vaccine to immunize specific pathogen-free (SPF) chickens, as a booster after prime with the existing 4–91/Mass vaccine. The animals were challenged with the IBV QX-like virus. The VLP-vaccinated birds produced S-protein-specific antibodies comparable to what is elicited by the existing vaccine, but with only a marginal increase after the boost. The detection of IBV RNA by qRT-PCR showed a reduction in viral shedding, and the effect of the vaccine on ciliary motility was also examined.

Finally, the use of duckweed as a host has also been explored by Tan et al. [102] as recently as 2025. The IBV antigen peptide EpiC was produced in duckweed at an estimated 3.84 µg/g fresh wt. and orally administered to chickens. This allowed the elicitation of the systemic IgG and mucosal sIgA response and 100% protection against challenge. The co-administration with IL-17B produced in duckweed as a type of adjuvant synergistically improved the immune response. The study demonstrated a reduced viral load in organs and protection against tissue damage with the duckweed-produced vaccine. By some measures, this prototype actually outperformed the existing H120 vaccine.

## 10. Plant-Produced Vaccines for Other Veterinary Coronaviruses

While the development of plant-produced veterinary coronavirus vaccines has focused on TGEV, PEDV, and IBV as described above, one additional study describes an innovative approach to a plant-produced vaccine for protection from porcine delta coronavirus (PDCoV), another common swine diarrheal disease. Ryu et al. [103] produced the S1 subunit of the spike protein in *N. benthamiana* in a fusion with lysine motifs (LysM) and coronin 1 coiled domains (ccCor1) to allow the display on the surface of *Lactococcus lactis* in a bacteria-like particle (BLP) formulation. The immunization of mice elicited an antibody response, but this was not characterized in detail.

## 11. Conclusions and Future Directions

The work described above provides convincing evidence that plants can produce efficacious vaccines against coronaviruses both for human and for veterinary use. The most widely studied platforms as it relates to coronavirus vaccines as well as some advantages and disadvantages are summarized in Table 2. Table 3 also includes the typical expression levels and approximate commodity costs for the raw material for different systems as well as a comparison of the potential output or yield for the major systems and a summary of the considerations for a scale-up. Numerous other thorough reviews have also compared different species used in plant pharmaceutical production and desirable characteristics for different applications [104,105,106,107,108]. While there are many diverse studies spanning over twenty years that are difficult to directly compare, one general trend is that efficacy can be increased by the fusion of the viral antigen to carrier proteins such as LTB, CTB, or Fc that may facilitate the targeting to appropriate cell types. For human disease, these vaccines can potentially provide low-cost oral delivery in resource-challenged parts of the world without reliance on cold chain or skilled medical personnel or the mobilization of additional supplies such as needles for administration. For veterinary use, orally delivered vaccines that could be incorporated into standard feeding practices instead of the injection of individual animals would be a great improvement and lead to a reduction in labor over current practices.

Proof-of-concept has been clearly demonstrated that production in plants can elicit a robust systemic and mucosal immune response, but, thus far, only the Medicago vaccine for SARS-CoV-2 and limited veterinary vaccines produced in tobacco for Newcastle disease in poultry, Classical Swine Fever (HERBAVAC^®^ CSF Green Marker), and Porcine Circovirus (HERBAVAC^®^ Circo Green) have been commercialized. Given the clear potential of the plant production of recombinant protein-based vaccines or other pharmaceuticals, the question is raised of what has hindered the further full implementation of this platform. These issues may be roughly divided into those intrinsic to the different plant species used as a platform and more general considerations related to plant production, industry adoption, intellectual property, or public relations considerations as discussed below.

Tobacco offers great advantages in speed in the case of transient expression and ease of manipulation of the pathways for post-translational modifications such as glycosylation. Clearly substantial advances are being made in additional modifications to optimize expression, protein processing, and folding that may still make tobacco-produced vaccines viable, but they will likely always require purification away from toxic compounds and may not be amenable to low-cost oral delivery in resource-challenged areas. The cost of raw materials is substantially higher than that for edible species without the potential for eliminating the cost of purification and downstream processing.

Other edible hosts such as potato, tomato, and lettuce have not achieved high enough expression levels to be effectively produced at scale and without having to consume an unrealistic amount of material. Tomato and lettuce are also highly susceptible to proper storage and potential spoilage if not used promptly. Rice potentially has great advantages for oral administration but still has some challenges in expression and is relatively labor-intensive for cultivation and may be less amenable to scale-up than some cereal species. The environmental cost in water usage is also significantly higher [109]. The commodity cost for rice is about twice that of corn as indicated in Table 3. Nevertheless, numerous prototype vaccines such as for Newcastle disease [110], classical swine fever [111], and *E. coli* [112], as well as other products, have progressed through promising preclinical work and, in some cases, into clinical trials. The MucoRice-CTB [113,114,115] cholera vaccine appears to be progressing through clinical trials and may be used soon but does not yet have regulatory approval. Other products such as human serum albumin from Ventria have been commercialized [116]. One additional potential advantage of rice is that it is typically self-pollinating, which reduces the chance of outcrossing to non-transgenic rice and may simplify regulatory issues.

**Table 3:** Comparison of parameters affecting use of plant expression systems.

Species	Expression Level	Cost per Metric Ton of Biomass	Yield (kg per Square Meter)	Scalability
Tobacco	Up to 110 mg/kg fresh wt. for coronavirus antigens [46], potential higher undisclosed proprietary information, reported at up to 5 g/kg fresh wt. for other proteins [117]	$6800 field grown, up to $20,000 greenhouse [118]	Up to 1.0–1.75 kg high density greenhouse [119]	Limited by building space and high infrastructure and purification costs, but biocontainment an advantage.
Maize	500 mg/kg seed [71]	$230 [120]	0.6–1.5 kg open field [121]	Very high up to 8,000,000-fold in a year with massive agricultural infrastructure.
Rice	0.282 g/kg seed dry wt. [60], other proteins ca. 1 g/kg [122]	$488 [123]	0.4–0.7 kg open field [124]	Very high and possible slight regulatory advantage due to self-pollination but slightly lower yield and may be limited by high water use.
Potato	55–163 ng/g fresh wt. [75]	$264 [125]	3.5–5.0 kg open field [126]	High-biomass but high water content and perishability impede scale-up, not amenable to consumption without cooking, and high water content and phenolics complicate downstream processing.
Tomato	Quantitation not done for coronavirus, up to 2 mg/kg fresh wt. for other proteins [127]	$104–150 [128]	4 to 10 kg field up to 100 kg for high-tech, climate-controlled commercial hydroponic systems [126]	High water content and diluted protein concentration require large processing volumes. High yield for field grown but limited by building space and high infrastructure and purification costs.
Lettuce	11.8 mg/g dry wt. [61]	$880 [129]	2–4 kg field up to 30 to 41 kg with continuous hydroponic or vertical farming methods [130]	Rapid growth and turnaround with low levels of secondary metabolite, but high water content of leaves leads to dilute target protein levels, high perishability, and need for immediate downstream processing.
Duckweed	3.84 ug/g fresh wt. [102]	$100 [131]	1.0–3.5 kg per day outdoor ponds, 10–20 kg per day in controlled experimental systems [132]	Very high and rapid growth and turnover but limited by building space for controlled indoor stacked systems.

Maize grain offers substantial advantages in allowing very high levels of expression and very rapid scale-up to high-level production on limited land without interfering with production for food and feed. The production of recombinant proteins in maize incorporated into the grain matrix allows for a form of bioencapsulation that stabilizes the protein in the digestive system in a manner similar to other nanoparticle approaches without additional cost or effort. Maize-produced vaccines can be administered as small and easily consumed tablets of pressed dried flour formulated with sugar for palatability or other excipients as necessary. Maize grain begins with a very inexpensive source for the production of proteins and has achieved some of the highest levels of recombinant protein accumulation up to several grams/kg seed. For a comparison to tobacco and rice, given the cost of the raw material (Table 3) and assuming an expression level of 1 g antigen per kg raw material in each case, this translates to a cost of the active ingredient of approximately $0.02/mg for tobacco and an exceptionally low cost of 100-fold less for maize and 50-fold less for rice [104]. Assuming an average of 10 mg antigen/g raw material, the cost of the other species studied would be approximately 100-fold higher. Scale-up for production for a major field grown crop such as maize is also much simpler than for most other recombinant protein production systems. As an example, assuming the current good expression levels of 1 g recombinant protein per kg seed and a yield of 4500 kg/acre, a typical small production farm of 160 acres (0.0002% of U.S. corn acres) can yield up to 720,000 thousand kg grain or 720 kg recombinant antigen/year. For an orally delivered dose of 1 mg that assumes no purification, this equates to 720,000,000 doses. If a 10 µg injected dose of purified antigen is used, this equates to a theoretical 72,000,000,000 doses. Assuming 70% recovery after processing and purification, this would still allow for up to 50,400,000,000 doses from a small farm. The material can be processed on the same site in a manner similar to other recombinant systems, allowing it to be kept under developer control with no need to have other commodity growers involved. This means that there is no imposition on the existing infrastructure [133]. Although potential cross-pollination to non-transgenic corn may potentially be a concern, this can be mitigated by maintaining a suitable distance from corn planted for food or feed purposes. Many improvements in all of the steps involved in recombinant protein production suggest maize as a practical and commercially viable production platform for viral antigens for vaccines for protection against coronaviruses both for current and future human needs as well as diseases of livestock.

One of the great advantages of plant production in general, relative to microbial systems such as *E. coli*, is the ability of plants to perform post-translational modifications such as glycosylation. However, there is a concern that the pattern of glycosylation in plants may affect the efficacy if it does not match that resulting from other systems. It is conceivable that differences in glycosylation patterns between plants and animals may affect the immunogenicity of plant-produced recombinant proteins. However, for some vaccines, there is also substantial evidence that efficacy is not that dependent on the specific pattern of post-translational modifications. At least seven cases have been documented in which subunit vaccine candidates subject to glycosylation have been expressed in plants and elicited strong immune responses, neutralization activity, protection on challenge, or correlates of protection. In addition to the work on hepatitis B as mentioned above, these examples include two influenza strains [134,135,136,137,138], rabies [139,140,141,142,143], TGEV [79], PEDV [71,90,91], Newcastle disease [144], and rotavirus [145,146,147]. However, as noted above, there can be effects on expression such as a loss of expression on the mutation of glycosylation sites [47] or effects on recombinant protein folding. Nevertheless, we are not aware of examples of plant-produced antigens that were expressed at a viable level and showed very poor immunogenicity. In many cases, substantial immunogenicity may be maintained with a plant glycosylation pattern while further optimization could be achieved with a more human-like glycosylation pattern. Continued work on modifications of other systems to produce proteins with a more human glycosylation pattern may further increase the efficacy for various applications.

The glycosylation pathway up through the endoplasmic reticulum is highly conserved between plants and animals and retention in the ER prevents proteins from entering the Golgi where the high mannose chains are trimmed and plant-specific glycosylation is added [148]. It has been previously demonstrated that ER targeting for recombinant proteins prevents plant-specific glycosylation and produces high-mannose-type glycosylation similar to that found in mammalian cells. In particular, the expression of an antibody directed to the ER in plants resulted in a glycoprotein with greater than 80% of higher mannose chains [149]. While there is typically a distribution in the carbohydrate length for any given site that cannot be precisely predicted, this suggests that the overall pattern in plant-produced antigens will be similar to that from mammalian cell production.

Concerns have been raised that the consumption of recombinant proteins with a plant glycosylation pattern could have unwanted immunogenic effects or cause an allergic reaction. There are numerous studies that show that plant-produced recombinant proteins can bind IgE and cause some interference with diagnostic tests, but the response is almost exclusively benign and causes no clinical symptoms [150,151,152,153]. Production in modified tobacco can indeed prevent the addition of xylose and fucose residues that may be of most concern. However, suitable subcellular targeting can also mitigate the presence of plant-specific glycosylation in other systems. Most humans consume significant amounts of plant material including proteins with plant glycosylation patterns on a daily basis, so the consumption of plant-produced proteins should not add undue increased risk.

In addition to the considerations discussed above, a number of other challenges can disrupt the path to commercialization for plant-produced vaccines or other pharmaceutical proteins [154,155]. Increasing expression levels will continue to be critical, as will optimizing scale-up and purification. Many potential products develop to the point of strong preclinical results but funding for the next steps of human clinical trials can be very difficult to obtain from venture capital, industry, or government sources. Large established companies as potential partners can be very reluctant to risk a new plant-produced pharmaceutical that has not already been through some human safety and efficacy trials or to seriously consider the full-scale implementation of a new system of production. There is also often a perception among potential partners of unclear intellectual property rights. Finally, public concerns regarding genetically modified organisms must be taken into account.

In conclusion, despite these numerous substantial bottlenecks, there is compelling evidence going back many years that plant-produced recombinant antigens can be produced in an efficacious form that provides protection from a pathogen challenge. Multiple different plant production systems may be viable for different purposes or different regions. The potential benefits of plant-produced vaccines, particularly oral vaccines that can be distributed and administered in resource-challenged areas without the constraints of traditional vaccines, remain substantial. Continued work is warranted along all the areas discussed above to increase expression, to optimize protein structure and processing, and to address less tangible issues such as general industry, investor, and public perception.

## Figures and Tables

**Table 1:** Human clinical trials with plant-produced prototype coronavirus vaccines.

Number	Sponsor	Phase	Summary
NCT04450004	Medicago	Phase I	Results published [18,19,20].
NCT04636697	Medicago	Phase 2/3	Results published [21,22].
NCT05065619	Medicago	Phase I/II in Japan	Terminated. (The study was terminated for ethical reason to prioritize the ancestral vaccination.)
NCT04894435	Canadian Immunization Research Network	Use as booster with varied injected vaccines	Still active.
NCT05197712	Baiya Phytopharm Vax-2	Phase I	Unknown status. (Study has passed its completion date and status has not been verified in more than two years.)
NCT05873374	Baiya Phytopharm Vax-2	Phase II	Unknown status. (Study has passed its completion date and status has not been verified in more than two years.)
NCT04953078	Baiya Phytopharm Vax-1	Phase I	Completed; no results posted.
NCT04473690	Kentucky Bioprocessing (Kbio) KBP-201	Phase I/II	Results posted, not published.

**Table 2:** Summary of plant systems used for prototype coronavirus vaccines ^1,2^.

	Species	Prototype Vaccines	Pros	Cons	Refs
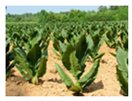	Tobacco	SARS, COVID-19, PEDV, TGEV	Speed and adaptability with transient expression; modification of glycosylation is possible but may not be critical for efficacy	Purification from toxic intermediates, higher cost, and instability of raw material	[14,15,16,17,18,19,20,21,22,23,24,25,26,27,28,29,30,31,32,33,34,35,36,37,38,39,40,41,42,43,44,45,46,47,48,49,50,51,52,53,54,55,56,57,74,80,81,82,83,84,85,86,87,88,89,90,91,92,93,94,95,96,97,98,100,101,103]
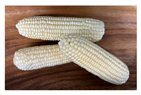	Maize	COVID-19, TGEV, PEDV	Seed-based, high expression, oral delivery, heat stable, long-term storage, low cost	Longer time to obtain initial transformed material	[71,76,77,78,79,90,91]
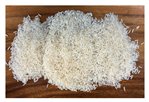	Rice	COVID-19, PEDV	Seed-based, oral delivery, heat stable, long-term storage	Labor-intensive cultivation	[58,59,60,93,97]
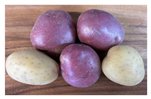	Potato	TGEV, PEDV	Edible for oral delivery but not palatable with the amount needed for dosing	Low expression, instability of raw material	[72,73,75,92,98,99]
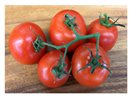	Tomato	SARS	Edible for oral delivery but not practical with the amount needed for dosing	Low expression, instability of raw material	[14]
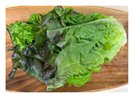	Lettuce	COVID-19, PEDV	Edible for oral delivery but not practical with the amount needed for dosing	low expression, instability of raw material, purification likely required, higher cost	[15,94,95]
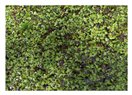	Duckweed	PEDV, avian infectious bronchitis virus	Rapid growth, minimal competition with food and feed production, FDA approval of duckweed-based protein powder for human consumption	Challenges with scale up for indoor production; more basic genome knowledge and resources needed	[96,102]

^1^ “Rows of Tobacco”: https://www.flickr.com/photos/universalpops/4951443515 (accessed on 1 June 2026) by David Hoffman is licensed under Creative Commons Attribution-NonCommercial-ShareAlike 3.0 Unported License. ^2^ “duckweed”: https://www.flickr.com/search/?text=duckweed&license=7%2C9%2C10 (accessed on 1 June 2026)—no known copyright restrictions.

## Data Availability

No new data were created or analyzed in this study. Data sharing is not applicable to this article.

## Abbreviations

The following abbreviations are used in this manuscript:

severe acute respiratory syndrome (SARS); Middle East respiratory syndrome (MERS); transmissible gastroenteritis virus (TGEV); porcine epidemic diarrhea virus (PEDV); infectious bronchitis virus (IBV); total soluble protein (TSP).
